# Identification of Common Blood Gene Signatures for the Diagnosis of Renal and Cardiac Acute Allograft Rejection

**DOI:** 10.1371/journal.pone.0082153

**Published:** 2013-12-16

**Authors:** Li Li, Kiran Khush, Szu-Chuan Hsieh, Lihua Ying, Helen Luikart, Tara Sigdel, Silke Roedder, Andrew Yang, Hannah Valantine, Minnie M. Sarwal

**Affiliations:** 1 Department of Pediatrics, Stanford University, Palo Alto, California, United States of America; 2 Division of Cardiovascular Medicine, Department of Medicine, Stanford University, Palo Alto, California, United States of America; 3 California Pacific Medical Center Research Institute, San Francisco, California, United States of America; Nazarbayev University, Kazakhstan

## Abstract

To test, whether 10 genes, diagnostic of renal allograft rejection in blood, are able to diagnose and predict cardiac allograft rejection, we analyzed 250 blood samples from heart transplant recipients with and without acute rejection (AR) and with cytomegalovirus (CMV) infection by QPCR. A QPCR-based logistic regression model was built on 5 of these 10 genes (AR threshold composite score>37% = AR) and tested for AR prediction in an independent set of 109 samples, where it correctly diagnosed AR with 89% accuracy, with no misclassifications for AR ISHLT grade 1b. CMV infection did not confound the AR score. The genes correctly diagnosed AR in a blood sample within 6 months prior to biopsy diagnosis with 80% sensitivity and untreated grade 1b AR episodes had persistently elevated scores until 6 months after biopsy diagnosis. The gene score was also correlated with presence or absence of cardiac allograft vasculopathy (CAV) irrespective of rejection grade. In conclusion, there is a common transcriptional axis of immunological trafficking in peripheral blood in both renal and cardiac organ transplant rejection, across a diverse recipient age range. A common gene signature, initially identified in the setting of renal transplant rejection, can be utilized serially after cardiac transplantation, to diagnose and predict biopsy confirmed acute heart transplant rejection.

## Introduction

Despite improvements in immunosuppressive therapy over the years, approximately 30–40% of heart transplant recipients require treatment for acute rejection (AR) in the first year after transplantation [1]. AR is a major risk factor for graft dysfunction, mortality, and the development of cardiac allograft vasculopathy (CAV) - the main cause of late graft failure [2]. Thus, methods that improve early diagnosis and treatment of AR are likely to reduce morbidity and improve survival after heart transplantation. Currently, the definitive diagnosis of allograft rejection relies on the endomyocardial biopsy (EMB)—an expensive, invasive, and inconvenient procedure. Most heart transplant recipients undergo routine EMB procedures up to 15 times in the first year, and more frequently if rejection is detected. This procedure however is limited by sampling error and inter-observer variability [3], [4]. Potential complications include arterial puncture, vasovagal reactions and prolonged bleeding during catheter insertion, arrhythmias and conduction abnormalities, pneumothorax, biopsy-induced tricuspid regurgitation, and even cardiac perforation [5]–[7]. A noninvasive biomarker panel for cardiac AR that allows frequent immunologic monitoring of the graft would be of considerable value [8], [9], and the diagnosis of AR prior to development of histopathological changes would enable the optimization of immunosuppressive therapy to prevent progression to chronic allograft dysfunction [10]. Recently our group has found a highly sensitive and specific gene-based biomarker panel for diagnosis and prediction of biopsy confirmed acute renal transplant rejection [11], which was independently validated in a randomized multicenter trial [12], [13]. In the previous study, we conducted extensive microarray discovery and QPCR validation studies on 489 unique peripheral blood samples from pediatric kidney transplant recipients, with and without biopsy proven AR. Correlation studies of gene expression profiles in peripheral blood samples of pediatric and young adult renal transplant patients with biopsy-proven acute rejection identified a highly regulated set of 10 genes by microarray analysis (CFLAR, DUSP1, IFNGR1, ITGAX, PBEF1, PSEN1, RNF130, RYBP, MAPK9, and NKTR), which was subsequently validated by QPCR, and which by logistic regression analysis yielded a probability score for non-invasive diagnosis of biopsy confirmed renal AR in pediatric and young adult patients [11].

Recent studies indicate that there likely is a common immunological mechanism for AR across different solid organ transplants [14]–[17]. We could define serum proteins highly increased in renal AR that were also increased during cardiac and liver AR [15]. The purpose of this study was to assess if the same peripheral blood gene panel discovered as pertinent for diagnosis of renal transplant rejection can also diagnose and predict heart transplant rejection in peripheral blood. We developed a 5-gene logistic regression model from our previously published 10-gene renal AR signature [11], that diagnosed acute cardiac allograft rejection in patient blood with 89% accuracy and predicted cardiac AR within 6 months prior to diagnosis by cardiac biopsy, with 80% sensitivity.

## Methods

### Study Design

**Figure 1:**
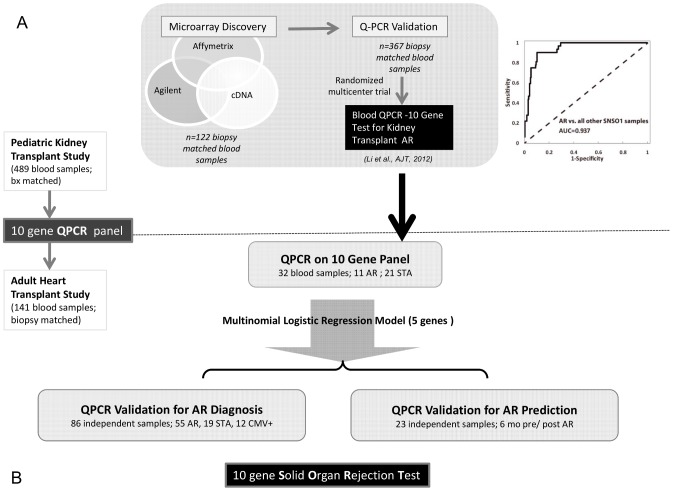
Study Design: A peripheral blood 10-gene panel for Solid Organ Transplant Rejection. **A.** The process of microarray discovery and QPCR validation of a 10 gene panel in 489 peripheral blood samples from pediatric and young adult renal transplant recipients, with validation of the gene biomarker panel in a prospective, randomized, multicenter trial (AUC = 0.937). **B.** The 10 genes were tested by QPCR in 141 peripheral blood samples from adult cardiac transplant recipients. A minimal logistic regression model of 5 genes was used for independent prediction for AR diagnosis in 86 samples and AR prediction prior to biopsy diagnosis.

The present study design is summarized in Figure 1 and described here: We previously conducted extensive cross-platform microarray discovery and QPCR validation studies on 489 unique peripheral blood samples from pediatric kidney transplant recipients with and without biopsy proven AR which led to the definition of a blood QPCR 10-gene signature (CFLAR, DUSP1, IFNGR1, ITGAX, PBEF1, PSEN1, RNF130, RYBP, MAPK9, and NKTR) for renal AR which by logistic regression yielded an AUC of 93.7% for diagnosis of AR using 5-genes (CFLAR, DUSP1, PBEF1, MAPK9, RNF130) in independent samples from a randomized multi-center trial (Figure 1A).

To investigate, whether the same 10-gene signature may also be modulated in cardiac AR, we investigated 141 peripheral blood samples with matched EMB, from adult heart transplant recipients by QPCR (Figure 1B). Firstly, we randomly assigned 32 samples into training (2/3) and test (1/3) sets for rejection and stable phenotypes; given the current clinical practice in most heart transplant centers of only treating Grade 3 AR, we included only rejection with Grade 3 in this QPCR discovery set. A multinomial logistic regression model using 5 genes, built in the training-set and validated in the test-set was secondly applied to an independent QPCR validation set of 86 blood samples with matched EMB. The model was tested (1) for its ability to segregate samples with AR from those without any evidence of rejection; it was tested (2) for its ability to discriminate AR from acute CMV infection in 12 blood samples from patients with documented active CMV infection; and the model was tested (3) for its ability to predict the development of CAV at 2- and 4-years post transplantation in patients with biopsy proven AR at 1 year, as AR is an important risk factor for the development of CAV. Finally, serial blood samples were available from 23 patients that were drawn within 6 months prior to or after an episode of biopsy-confirmed AR. The 5 gene model was tested in this QPCR prediction-set to ascertain the “rejection score”, to determine whether the gene expression score rose prior to episodes of biopsy-proven AR, and whether the score declined after treatment of the rejection event.

### Study Population

#### Ethics Statement

All patients involved in this study provided informed consent to the study protocol approved by the institutional (Stanford University) review board for studies in human subjects.

#### Sample Selection

This biomarker study utilized a cohort of 45 consecutive patients undergoing first heart transplantation between January 2002 and May 2005 at Stanford University. This cohort was a selected subset of samples from a clinical trial that was funded by the National Institutes of Health (Program Project Grant (PPG) 5P01AI050153-02) and had been assembled prospectively to study the relationship between CMV infection and the development of CAV. Exclusion criteria for this trial included age <10 years, renal dysfunction requiring prolonged dialysis, and inability or unwillingness to provide signed informed consent. Study patients in this trial had been monitored for acute cellular rejection by surveillance EMB performed at the following scheduled intervals after transplant: weekly during the 1st month, biweekly until the 3rd month, monthly until the 6th month, and then at months 9 and 12. Biopsies were graded according to the 1990 International Society for Heart and Lung Transplantation (ISHLT) classification system as 0, 1A, 1B, 2, 3A, and 3B (Table 1) [18]. Though there was a large pool of samples in the original clinical trial (PPG 5P01AI050153-02), a modest number of samples could not be used as not all samples had adequate RNA remaining after conduct of the CMV studies from the original grant, some were currently being utilized as part of concomitant ongoing studies by HV, and samples were not selected if the amount of remaining RNA in the archive was <500 ng. In addition, to maintain strict quality controls for the QPCR experiments, only those samples were selected that had excellent quality RNA (RIN>5). Final sample selection for this study used all remaining samples and then further sub-selected samples that met the following clinical phenotypes: (1) acute rejection, CMV− (acute rejection or AR group); no rejection, CMV− (stable or STA group); no rejection, CMV+ (CMV group); (2) AR blood samples were required to be drawn on the same day of the biopsy, just prior to the biopsy procedure and prior to any treatment intensification for AR; (3) STA patients selected were demographically matched with the identified AR patients. In addition, (4) for all selected AR samples, we pulled all available samples paired with these rejection episodes within a 6 month time frame prior to (pre-) and after (post-) the rejection episode. The rationale for this aspect of sample selection before and after AR was based on our previous study on kidney transplant rejection that suggested that the rejection gene signature in kidney transplantation could identify pre-acute rejection samples within a 6 month time-frame prior to AR [11]–[13]. (5) Multiple samples from a single patient were utilized as long as they had a matched biopsy with conclusive phenotypic diagnosis of AR or STA, with the caveat that the STA sample had to be >1 year distant from the AR episode, so that there was no overlap between STA and pre- and post-AR samples which were only collected within the 6 month timeframe of AR. This resulted in a final selection of a total of 141 unique blood samples selected from 45 unique adult heart transplant recipients. The breakdown of the different blood sample categorizations were as follows: 40 samples were selected where the EMB showed no evidence of cellular rejection (Grade 0), 31 samples were selected where the EMB was classified as Grade 1A, 22 samples were selected where the EMB was classified as Grade 1B, only 2 samples were available when the EMB was classified as Grade 2, and 11 samples were selected where the EMB was classified as Grade≥3A. All available Grades of AR meeting our selection criteria were selected for this analysis. In addition, 12 blood samples were selected as they had been drawn during episodes of CMV reactivation (defined as >100 copies of CMV DNA amplified from peripheral blood mononuclear cells), and 23 samples were drawn within 6 months prior to (n = 11), or after an episode of cellular rejection (n = 12). For the purposes of this study, stable (STA) was defined as the EMB showing no evidence of lymphocytic infiltrate (Grade 0), while acute rejection (AR) was defined as EMB showing evidence of mild-severe lymphocytic infiltrate (Grade 1A–3B).

**Table 1:** 1990 ISHLT Standardized Cardiac Biopsy Grading Scheme for Acute Cellular Rejection and Corresponding Number of Samples Studied [18].

Grade	N = 141	Histological features
**0**	75 (40+23*+12**)	No rejection
**1, mild**	53	
A- Focal	31	Focal perivascular and/or interstitial infiltrate without myocyte damage
B- Diffuse	22	Diffuse infiltrate without myocyte damage
**2, moderate (focal)**	2	One focus of infiltrate with associated myocyte damage
**3, moderate**	11	
A-Focal	7	Multifocal infiltrate with myocyte damage
B- Diffuse	4	Diffuse infiltrate with myocyte damage

23 samples drawn within 6 months prior to or after episodes of acute rejection

12 samples drawn from patients with CMV infection (>100 copies of CMV DNA amplified from peripheral blood mononuclear cells).

In the study, yearly coronary angiograms were performed with intravascular ultrasound (IVUS), enabling highly accurate measurements of vessel wall thickness, for assessment of CAV which is characterized by diffuse intimal thickening of the graft coronary arteries [19]. All study participants were assigned a CAV score from 0–4: 0 = no evidence of CAV by angiography or IVUS; 1 = coronary artery intimal thickening by IVUS without angiographic disease; 2 = coronary artery stenosis<30% by angiography; 3 = coronary artery stenosis of 30–70% by angiography; 4 = coronary artery stenosis>70% by angiography or placement of an intra-coronary stent.

Peripheral whole blood samples were collected and stored at the following time-points post-transplant in the parent PPG: day 14; months 1, 2, 3, 4, 5, 6, 9, 12, 16, 20, 24, 28, 32, 36, 40, 44, 48, 52, 56, and 60. For demographic characteristics of patients included in the study refer to Table 2.

**Table 2:** Clinical profile of 45 study patients.

*Patient Clinical Variables*	
Age (years, mean ± SD)	48.2±17.3
Sex (% male)	73%
Race/ethnicity, n (%)	
Caucasian	36 (80%)
-Asian	1 (2%)
-Hispanic	4 (9%)
-African-American	3 (7%)
-Other	1 (2%)
Primary disease, n (%)	
-Ischemic CM	16 (36%)
-Dilated CM	58%)
-Other	3 (7%)
Diabetes, n (%)	13 (29%)
Hypertension, n (%)	45 (100%)
History of Smoking, n (%)	7 (16%)
Sample time (mean ± SD) [months post Txp.]	15.0±10.9

#### Patient Immunosuppression Protocol

Post-transplant immunosuppression consisted of Daclizumab (1 mg/kg IV) administered at the time of transplant surgery and on alternate weeks for a total of five doses, Cyclosporine (3–5 mg/kg/day); Prednisone initiated at 1 mg/kg/day and tapered to <0.1 mg/kg/day by the 6th post-operative month; and either Mycophenolate mofetil 1000–3000 mg daily, or Sirolimus 1–4 mg daily. Changes to this standard immunosuppressive regimen were made on an individual basis. All patients in whom either donor or recipient was CMV antibody positive received standard CMV prophylaxis consisting of 4 weeks of intravenous Ganciclovir. Those recipients who were CMV antibody negative and received a heart from a CMV antibody positive donor received an additional 3 months course of CMV hyperimmune serum and up to 80 days of Valganciclovir.

### Sample Collection, Total RNA Extraction and Quantitative Real-time PCR (QPCR)

Peripheral blood (2.5 mL) was collected into PAXgene™ Blood RNA tube (PreAnalytiX/Qiagen, Valencia, CA, USA) containing lysis buffer and RNA stabilizing solution. Total RNA was extracted with the PAXgene™ Blood RNA System (PreAnalytix/Qiagen, Valencia, CA, USA) following the manufacturer's instructions and as previously published [11], yielding a final concentration of 50–300 ng/µl. A total of 500 ng RNA were reverse transcribed in a 20 µl reaction using the RT^2^ First Strand cDNA Synthesis Kit (Bioscience), followed by quantitative real-time polymerase chain reaction (QPCR) in 384-well plates using the QPCR Master Mix (RT^2^ SYBR Green/ROX) (Bioscience). 5 ng cDNA were added to each 10 µl QPCR reaction in duplicated wells. QPCR reactions were run on the ABI PRISM 7900HT Sequence Detection System (Applied Biosystems, Life Technologies, Foster City, CA). The relative amount of RNA expression was calculated using comparative C_T_ method [20] with ribosomal 18S RNA as endogenous control gene and universal RNA as reference sample (Human Universal RNA, Stratagene, Agilent Technologies, Santa Clara, CA). Additionally, FOXP3 a previously reported AR biomarker, was included in each plate to serve as a positive control gene.

### Selection of 10 genes for QPCR in heart transplantation

Selection of the 10 genes for gene expression analysis in this study was done through a multi-platform microarray discovery followed by QPCR validation in kidney transplantation [11]. Among 10,412 common genes probed on all the platforms analyzed 32 genes were selected based on FDR of <5% for differential expression in AR and biological relevance to the immune response; this resulted in a selection of 32 genes [11]. QPCR validation on an independent set of samples resulted in 10 genes significantly differentially expressed between rejection and stable graft groups which were subsequently used for building a classification model by logistic regression [11]. In the present study, the same set of 10 genes (CFLAR, DUSP1, IFNGR1, ITGAX, PBEF1, PSEN1, RNF130, RYBP, MAPK9, and NKTR) was investigated in heart transplant blood samples by QPCR.

### Statistical Analysis

Mean ± standard deviations of were calculated for patient demographic variables, and mean ± standard errors of means were determined for QPCR results. Student T-tests, Chi-square tests, Hypergeometric tests [21], [22], Spearman-, Pearson-, or Kendall- correlation coefficients, and logistic regression models were calculated using Statistical Analysis Software (SAS) Version v9.2 (SAS Institute Inc., NC) and R version 2.15. A 5-gene logistic regression model was built on the categorical variables AR versus STA using relative gene expression values. The model was built in SAS 9v.2 and reproduced in R 2.15, with likelihood p-value = 0.008. P-values were two-sided, and those ≤0.05 were considered significant in all statistical tests. We used Pearson correlation coefficients to evaluate the potential association between continuous variables and gene expression of the 5 genes from QPCR and used T-tests to evaluate gene expression levels for the categorical variables, such as recipient and donor gender. We determined whether a high peripheral gene-based prediction score for cardiac AR predicted the subsequent development of CAV by calculating Spearman correlation coefficients between the gene-based probability scores for AR and subsequent CAV scores. We used the hypergeometric test [21] to determine whether the proportion of the highly expressed genes in each cell type was statistically significant or not. The p-values from hypergeometric tests were corrected for multiple hypotheses using Benjamini–Hochberg correction [22].

## Results

### 5 genes diagnosed Acute Cellular Rejection after Heart Transplantation in Blood

**Figure 2:**
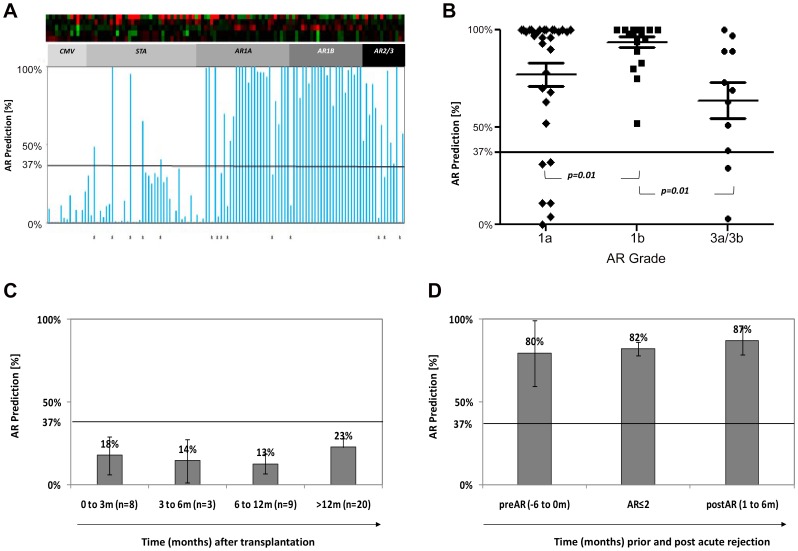
Predicted Probability of AR in 141 Peripheral Blood samples from Adult Heart Transplant Recipients. **A.** The predicted probability of a sample having a non-invasive diagnosis of AR, based on the logistic regression score on the 5-gene model is shown on the Y Axis (score range 0–100%). A score>37%, from the model, classifies a sample as AR, a score <37% from the model classifies a sample as non-AR. The score is shown on all 141 samples, inclusive of the training (n = 32; 11 Grade 3 AR, 21 STA) and the test set samples (12 CMV, 19 STA, 31 AR-Grade 1a, 22 AR-Grade 1b, 2 AR Grade 2). The clinical sample phenotype is based on the matched biopsy histology read. The misclassified samples from the histology read and the blood gene-model read are marked by asteryx. **B.** The Individual and group predicted probabilities for all 66 AR samples. The blood-gene model classifies all AR-Grade 1b correctly (a significant finding with p = 0.01, for classification of other AR grades). **C.** The predicted probabilities for AR for all Stable samples without any evidence of acute rejection (STA), with sampling times at different times post-transplantation. **D.** The predicted probabilities for AR for all 55 untreated AR samples (AR-Grades≤2), where no treatment intensification was given for the diagnosis of AR. Serial samples from these patients collected within 1–6 months prior (n = 11), or within 1–6 months after (n = 12), these AR episodes. The gene-model predicts AR prior to biopsy diagnosis and remains elevated in most samples without immunosuppression intensification.

QPCR-generated gene expression data for a set of 10 genes (CFLAR, DUSP1, IFNGR1, ITGAX, PBEF1, PSEN1, RNF130, RYBP, MAPK9, and NKTR), originally identified and validated in 458 peripheral blood samples from pediatric recipients of a renal transplant [11], were cross-validated in peripheral blood samples from 141 heart transplant recipients and demonstrated significant differences between rejection and non-rejection groups. Using only rejection with Grade 3 in the discovery set and by randomly assigning STA samples, a logistic regression model was built in the 1/3 training-set alone to predict AR in the independent 2–3 validation-set set. Using a multinomial logistic regression model, a minimum set of 5 genes was identified that could accurately classify acute rejection blood samples from samples without acute rejection (stable, STA) with a median accuracy of 0.73. The model from the published kidney 5 genes (DUSP1, MAPK9, NKTR, PBEF1, and PSEN1) did not achieve better performance than the best subset of the 5 genes selected in the heart data-set (DUSP1, IFNGR1, MAPK9, PBEF1, and RYBP) which had with a chi-square score of 9.57. Chi-square score for logistic regression models built using the 10 genes showed that in the data-set used, using 5 gene models had the same performance as models using six or more genes (Chi-square of the 5 genes and 10 genes are 9.57 vs. 9.79 respectively). Based on the Receiver Operating Characteristic (ROC) curve with an AUC of 0.89 (Figure 2A), the cutoff for the predicted probability for a sample to be classified as AR (Theta = θ) was θ = 0.37 which had the best sensitivity and specificity by maximizing the correct rate, to discriminate between AR and STA. In the logistic regression, each of the 5 regression coefficients describes the size of the contribution of that gene as a risk factor for diagnosing AR, where the larger the coefficient, the greater the influence of that gene in AR. A positive coefficient suggests that the explanatory variable increases the probability of AR where as a negative coefficient decreases the probability of AR.

The fixed 5 gene-model was subsequently tested in 86 independent samples with varying AR grades, STA and CMV diagnoses, and identified AR samples of all grades <3 with 89% accuracy (87% sensitivity, 90% specificity is, 94% PPV, 80% NPV; Figure 2A, misclassified samples in 2A are indicated by an asterix; Table 3 shows individual prediction scores for the different AR grades). The sensitivity for prediction of AR was highest for samples with ISHLT biopsy Grade 1B (100%), and was 82% for prediction of Grades 3A/B and 81% for Grade 1A. Sensitivity for prediction of ISHLT Grade 2 events was not calculated as there were only 2 samples in this category and both classified correctly. The 5-gene prediction score could not segregate samples with paired biopsies with fibrosis (Grade 3B; p = 0.21) and myocyte damage (Grades 3A and 3B; p = 0.07) from those with lesser grades of AR (Grades≤2). The prediction probability of the 5-gene model was highest in blood samples from Grade 1B rejection (Grade 1B vs. Grade 1A; p = 0.01; Grade 1B vs. Grade 3A/B, p = 0.01), which allows to hypothesize that the signal for the 5-gene expression profile, likely comes from trafficking mononuclear cells in blood, as previously suggested in renal rejection [11], [23], and reflects the extent of the inflammatory response in the graft, which is known to be greatest in Grade 1B AR (Figure 2B).

**Table 3:** Prediction Performance of the Gene-Model on Different Clinical Phenotypes (Biopsy Confirmed).

Prediction Sets	AR (prediction)	STA (prediction)	Total	Sensitivity (AR) or Specificity (Non-AR)
**AR (N = 55)**	49	6	55	89% Sensitivity
-A (N = 31)	25	5	31	81% Sensitivity
-1B (N = 22)	22	0	22	100% Sensitivity
-2 (N = 2)	1	1	2	Not calculated
**Non-AR (N = 31)**	3	28	31	90% Specificity
-STA (N = 19)	3	16	19	84% Specificity
-CMV+(N = 12)	0	12	12	100% Specificity

*For all 86 samples in the prediction set, Sensitivity = 87%, Specificity = 90%.*

**AR**: acute rejection (Grades 1–3); **STA**: stable (Grade 0).

### The 5-gene model discriminated AR from active CMV

All 12 CMV-positive samples were correctly predicted to have no AR, suggesting that there is no concern for innate immune activation in CMV confounding the 5-gene expression panel for AR in blood.

### The 5-gene AR Prediction Score was significantly associated with development of CAV

As cardiac AR is a known important risk factor for the development of CAV the second most common cause of death, early prediction of CAV in patients with AR would be of great value. In this regard, we investigated whether our 5-gene model was associated with the development of CAV in patients with AR. There was a significant positive correlation between the probability score for prediction of AR in a blood sample drawn at 1 year post-transplantation, and the subsequent development of CAV in that same patient at 2 years (r = 0.73, p = 0.02) and at 4 years (r = 0.82, p = 0.01) post-transplantation. Furthermore, predicted probabilities of AR at 1 year were significantly higher in patients with higher grades of CAV (CAV score≥3) vs. mild grades of CAV (CAV score≤2) at 4 years post-transplantation (99%±1% vs. 32%±14%, p = 0.001), which indicated that patients with higher predicted AR probability, independent of AR histology grade, may be at greater risk to develop more severe CAV at subsequent follow-up.

### The 5-gene model was not confounded by demographic or clinical variables

No significant demographic or clinical variables (including age, sex, and time post-transplant) were found to be confounders for the ability of the 5-gene model to diagnose AR (maximum |r|<0.4 or p>0.05), and specifically time-post transplant for sampling did not confound the score, which has been an issue in other biomarker studies of this nature [3].

### The 5-gene model predicted AR in blood *prior* to histological diagnosis in EMB

The AR prediction score was measured on blood samples drawn within a period of 6 months prior to a biopsy proven AR event (grades 1A, 1B, or 2; Figure 1). As seen in Figures 2C and 2D, there was a statistically higher likelihood (p<0.0001) of a high prediction score for AR (mean prediction score 80%; Figure 2D) in the blood samples drawn prior to AR than a blood sample drawn prior to a negative biopsy (mean prediction score 17%; Figure 2C). The 5-gene probability score for AR in many blood samples drawn within 1–6 months *after* treatment of acute rejection varied between (0%–100%), with an average prediction score of 87% (n = 12 samples; Figure 2D).

## Discussion

We conducted the first study to cross-validate a gene expression panel that detected acute rejection after kidney transplantation for detection and prediction of acute rejection in heart transplant recipients. Our 10-gene panel was differentially regulated in the periphery at the time of histologically confirmed acute rejection irrespective of tissue source. Additionally, these genes were indicative of histological acute rejection in both children and adults, as our study in renal AR [11] was performed in pediatric and young adult renal allograft recipients and the present study in cardiac AR was performed in adult heart transplant recipients. It was possible to narrow the original 10-gene panel to an even smaller set of 5-genes that were not confounded by clinical variables, such as transplant recipient age and sex, time post-transplant, or innate immune activation, discriminating AR from concomitant CMV infection. The lack of any confounding effect from active CMV infection suggested that the gene expression signature reflects the identification of a specific alloimmune trafficking response that is independent of the heightened innate immune response seen in CMV infection.

This peripheral blood gene expression signature correlated with the activation profile of the inflammatory infiltrate, rather than the grade of rejection or the extent of fibrosis or myocyte damage. The 5-genes have been shown previously to be highly expressed in cells of the monocyte and macrophage lineage [11], [23], suggesting that the gene expression panel is detecting trafficking of activated monocyte lineage cells; cells that are common to the inflammatory injury of acute rejection in kidney and heart transplantation. Individual genes such as CD27, CD40, TIRC7, cytokines (interferon-γ, interleukin [IL]-2, IL-4, IL-6, IL-8), and cytotoxic T-cell effector molecules (Perforin, Granzyme B, FasL) have been previously found to be elevated in rejecting biopsy samples [24]–[30] and peripheral in blood [31]–[33] at the time of cardiac allograft rejection, but many of these are also regulated during infection and other causes of inflammation. Microarray technologies offer the option of simultaneously screening thousands of novel candidate genes in an unbiased fashion, while controlling for multiple clinical confounders, enabling the identification of panels of genes in peripheral blood that may be very sensitive and specific for histological acute rejection [34], [35] and provide more robust performance than any single gene analysis [3], [36]. The discovery of the 10 gene-set in this study came from global gene expression analysis of ∼54,000 genes on different microarray platforms using peripheral blood samples from pediatric kidney transplant recipients [11] and this gene-panel was validated as highly accurate for acute rejection diagnosis in a prospective, randomized multicenter clinical trial. As the same genes were found to also detect AR in adult heart transplant recipients in the present study, the performance of this gene-set to detect biopsy confirmed AR in different solid organs and across the span of gender, post-transplant time, differences in immunosuppression, transplant centers and recipient age, is highlighted and further supports the presents of a common rejection specific immune axis.

The Cardiac Allograft Rejection Gene expression Observational (CARGO) study [3], identified an 11-gene PCR classifier, largely from the literature, that was subsequently commercialized into the AlloMap Molecular Expression Test (XDx, Brisbane, CA). This test provides a negative predictive value (NPV) of 99% for moderate-severe cellular rejection by EMB, providing a means for *ruling-out* the presence of rejection but has low positive predictive value (PPV) and sensitivity for *ruling-in* the presence of AR. The clinical utility of a blood gene profiling approach for *ruling out* AR was demonstrated in a randomized study on 600 heart transplant recipients, where there was non-inferiority of an Allomap-based rejection monitoring strategy, compared to EMB, with respect to a composite endpoint of AR, graft failure and death, and a reduction in the number of EMBs performed in this study by almost 70%, consistent with the high NPV associated with the Allomap test [37]. However, the PPV of 20–40% for the Allomap test for *ruling-in* the presence of AR in the same study suggested that complementary approaches for the diagnosis and prediction of AR, such as the use of the present 5-gene panel by this study, are needed.

Although management of heart transplant recipients often varies between centers, most transplant programs only consider rejection of Grade 3A or 3B (showing myocyte damage) as clinically relevant, and therefore warranting treatment. Currently, AR of grades of 1A, 1B and 2 are frequently dismissed, without any additional treatment delivery, perhaps because these lower histological grades of rejection are observed so commonly in the protocol biopsies performed. Interestingly, the inflammatory infiltrate that is common to all histological grades (1–4) of AR and is singularly absent in the non-rejection biopsies (Grade 0), suggests that the presence of an infiltrate is a very common finding, and in the absence of myocyte damage its clinical relevance in heart transplantation remains unclear. Nevertheless, the presence of an inflammatory infiltrate of predominantly mononuclear cells is the hallmark of AR in other solid organ transplants such as kidney [38], lung [39] and small intestine [40], where the infiltrate is believed to be pathologically and clinically relevant, and triggers a treatment response of bolus immunosuppression. The ISHLT 1990 classification scheme for acute cardiac allograft rejection distinguished 3 grades of mild-moderate cellular rejection: Grades 1A, 1B, and 2, based on absence (Grades 1A and 1B) or presence of myocyte damage (Grade 2), and focal (Grade 1A) versus diffuse (Grade 1B) nature of the lymphocytic infiltrate (Table 1). Subsequent clinical investigations of these mild-moderate rejection grades focused on their temporal occurrence, requirement for therapy, and progression to more severe grades of rejection [41]–[45], and ultimately led to a revision of the ISHLT classification scheme in 2004, which included a single mild grade of rejection (1R), which subsumed the original Grades 1A, 1B, and 2 [46].

The 5-gene model developed and tested in this study can diagnose acute cardiac rejection of Grades 1A–3B (no Grade 4 samples were available for this study), with the highest confidence for diagnosing Grade 1B rejection. Molecular subtyping has demonstrated evidence of myocyte apoptosis in Grade 1B biopsies that is a feature of myocyte damage typical of Grade 3A biopsies, but not of less severe (Grade 1A) rejection [47]. Such data suggests that Grade 1B biopsies may share molecular similarities with Grades≥3A, and that molecular approaches may provide novel insights into tissue injury that may complement the light-microscopic criteria traditionally used for biopsy grading. Bernstein *et al* [48] recently performed a *post hoc* analysis of the CARGO data, specifically examining gene expression scores for blood samples accompanying EMB of varying grades. They demonstrated that the mean gene expression scores for Grades 1B and ≥3A were indistinguishable, once again suggesting a potential overlap along a molecular spectrum of rejection severity. A recent study by Holweg et al. [49] profiled EMB of patients with different cardiac transplant rejection grades. Although grade 1B was found to be distinct from the clinically relevant AR grades 3A and 3B in this study, all of these grades were found to share a number of overlapping pathways consistent with common physiological underpinnings. The mean gene expression score for Grade 1B also suggested its molecular distinction from other Grades (1A and 2) classified as mild rejection in the 2004 revised grading scheme [46]. Our results are consistent with those of Bernstein, and suggest that combining Grades 1A, 1B, and 2 in the 2004 revised grading scheme may undermine the independent value and distinct inflammatory nature of different rejection grades. The gene expression similarities identified here in grade 1B and grade 3 AR have the potential to revise the clinical perspective on acute graft rejection, pending the results of additional prospective studies.

The 5-gene model developed in this study could also *predict* the onset of AR, months before EMB based histological diagnosis. Importantly, the AR probability score defined by our 5-gene model decreased again after augmented immunosuppressive therapy in patients with rejection grades 3A/B, and remained elevated in untreated cases of AR of grades≤2. Further studies are required to evaluate the prediction probability of this gene-set as a means to titrate immunosuppression in heart transplantation, without the need for frequent protocol biopsies.

Our previous work in kidney transplantation [11], [23], [34] has highlighted the fact that the 10 selected genes in our original model are highly expressed in cells of the monocyte lineage. The statistical approach of deconvolution [23], now available as cell-specific Significance Analysis of Microarrays or cSAM [50], also demonstrated that the monocyte-specific signal in peripheral blood [11], [23] drives the differential expression of peripheral genes in acute renal transplant rejection. As our previous studies in kidney transplant rejection [23] have not identified any differences in the numbers of circulating monocytes, the gene signature likely reflects an activation status of this cell lineage, though additional work is required to validate these findings as sorted monocytes were not available for evaluation in this study. As this same gene set also displayed differential regulation in all grades of acute heart transplant rejection, our work likely highlights a novel, and hitherto unrecognized role for the activated monocyte as the key peripheral trafficking cell in acute rejection, both within the graft and as a biomarker for acute rejection in the periphery. Our group is currently assessing the performance of the same gene set as a non-invasive marker for AR in other solid organ transplants, specifically lung and intestinal transplantation.

Although these results are intriguing, they mandate further validation in larger, prospective cohorts, as the sample set in this study was small and the analysis was retrospective in nature. The gene panel demonstrated the utility of distinguishing the presence of cellular rejection (graded from 1 to 3, or mild to severe) from immunologic quiescence; however, further refinement of our algorithm is planned to distinguish clinically-relevant grades of rejection from those that do not require treatment. Additionally, this study focused on the ability of our model to diagnose acute cellular rejection, and not antibody-mediated (humoral) rejection, as diagnostic assays for the presence of antibody-mediated rejection were not routinely performed during the sample collection period.

CAV, the leading cause of late morbidity and mortality after heart transplantation, is a complex multifactorial process mediated by both immune and non-immune factors. The diffuse nature of CAV, which usually involves the entire coronary arterial tree [51] suggests primarily an immune etiology. Prior observational studies suggest that cellular AR and CAV are closely related processes [52], [53]. Our finding of a positive association between AR prediction scores and subsequent development of CAV further supports this theory. A similar finding was also noted by the an association of the AlloMap with cardiac vasculopathy, as a higher AlloMap score was found in 20 cardiac recipients with EMB confirmed vasculopathy and compared to 49 control patients [54]. Thus our finding also supports that gene expression testing could be used to determine a patient's future risk of CAV—and could be used to potentially tailor prophylactic strategies to prevent CAV development. Additional validation of this work in larger cohorts is warranted. The strong correlation seen for the AR prediction score of the current 5-gene model with the development of subsequent CAV suggested that this inflammatory infiltrate, even independent of rejection grade and similar to its downstream effect in other solid organs [11], [36], [37] may not be benign and likely accelerates the evolution of chronic injury, and is therefore potentially deserving of clinical vigilance and treatment.

In conclusion, an internally validated 5-gene classifier panel, from a larger set of 10 genes, has been developed to non-invasively screen for the presence of acute cellular rejection after heart transplantation; the same 10 genes also being diagnostic of acute kidney transplant rejection. The markers studied had *a priori* plausibility, given their demonstrated utility for diagnosis of acute rejection after kidney transplantation, reflecting common pathways of immune activation [17]. The high specificity and PPV of the 5-gene panel in peripheral blood samples fulfill a critical unmet need for AR monitoring in heart transplantation and warrant additional validation. As mentioned previously, the currently-available AlloMap test has very high NPV, and therefore enables clinicians to *rule out* the presence of rejection. This assay, with a high PPV, would therefore be complementary by concurrently enabling clinicians to *rule in* the presence of rejection and additionally *predict* a risk-read out for AR prior to any clinical graft dysfunction. A strategy that combines both non-invasive tests could therefore enable biopsy avoidance in a larger number of patients than either test alone. The observed gene expression patterns in this study challenge the current paradigm of classifying certain rejection grades, such as Grade 1B, as “mild” and therefore not requiring intensification of immunosuppressive therapy. Further testing in larger patient numbers and prospective clinical trials will be necessary to additionally validate this panel, with the goal of developing a noninvasive and clinically relevant test for diagnosis of cardiac allograft rejection.

## References

[1] Taylor DO, Stehlik J, Edwards LB, Aurora P, Christie JD, et al (2009) Registry of the International Society for Heart and Lung Transplantation: Twenty-sixth Official Adult Heart Transplant Report-2009. J Heart Lung Transplant 28: 1007–1022. 19782283 10.1016/j.healun.2009.08.014

[2] Raichlin E, Edwards BS, Kremers WK, Clavell AL, Rodeheffer RJ, et al (2009) Acute cellular rejection and the subsequent development of allograft vasculopathy after cardiac transplantation. J Heart Lung Transplant 28: 320–327. 19332257 10.1016/j.healun.2009.01.006

[3] Deng MC, Eisen HJ, Mehra MR, Billingham M, Marboe CC, et al (2006) Noninvasive discrimination of rejection in cardiac allograft recipients using gene expression profiling. Am J Transplant 6: 150–160. 16433769 10.1111/j.1600-6143.2005.01175.x

[4] Wong BW, Rahmani M, Rezai N, McManus BM (2005) Progress in heart transplantation. Cardiovasc Pathol 14: 176–180. 16009314 10.1016/j.carpath.2005.05.001

[5] Baraldi-Junkins C, Levin HR, Kasper EK, Rayburn BK, Herskowitz A, et al (1993) Complications of endomyocardial biopsy in heart transplant patients. J Heart Lung Transplant 12: 63–67. 8443204

[6] Deckers JW, Hare JM, Baughman KL (1992) Complications of transvenous right ventricular endomyocardial biopsy in adult patients with cardiomyopathy: a seven-year survey of 546 consecutive diagnostic procedures in a tertiary referral center. J Am Coll Cardiol 19: 43–47. 1729344 10.1016/0735-1097(92)90049-s

[7] Navia JL, Atik FA, Vega PR, Garcia M, Starling RC, et al (2005) Tricuspid valve repair for biopsy-induced regurgitation in a heart transplant recipient. J Heart Valve Dis 14: 264–267. 15792190

[8] Evans RW, Williams GE, Baron HM, Deng MC, Eisen HJ, et al (2005) The economic implications of noninvasive molecular testing for cardiac allograft rejection. Am J Transplant 5: 1553–1558. 15888068 10.1111/j.1600-6143.2005.00869.x

[9] Mehra MR, Feller E, Rosenberg S (2006) The promise of protein-based and gene-based clinical markers in heart transplantation: from bench to bedside. Nat Clin Pract Cardiovasc Med 3: 136–143. 16505859 10.1038/ncpcardio0457

[10] Kienzl K, Sarg B, Golderer G, Obrist P, Werner ER, et al (2009) Proteomic profiling of acute cardiac allograft rejection. Transplantation 88: 553–560. 19696639 10.1097/TP.0b013e3181b119b1

[11] Li L, Khatri P, Sigdel TK, Tran T, Ying L, et al (2012) A peripheral blood diagnostic test for acute rejection in renal transplantation. Am J Transplant 12: 2710–2718. 23009139 10.1111/j.1600-6143.2012.04253.x PMC4148014

[12] Sarwal MM, Ettenger RB, Dharnidharka V, Benfield M, Mathias R, et al (2012) Complete Steroid Avoidance Is Effective and Safe in Children With Renal Transplants: A Multicenter Randomized Trial With Three-Year Follow-Up. Am J Transplant 12: 2719–2729. 22694755 10.1111/j.1600-6143.2012.04145.x PMC3681527

[13] Naesens M, Salvatierra O, Benfield M, Ettenger RB, Dharnidharka V, et al (2012) Subclinical Inflammation and Chronic Renal Allograft Injury in a Randomized Trial on Steroid Avoidance in Pediatric Kidney Transplantation. Am J Transplant 12: 2730–2743. 22694733 10.1111/j.1600-6143.2012.04144.x PMC3459071

[14] Wang E, Worschech A, Marincola FM (2008) The immunologic constant of rejection. Trends Immunol 29: 256–262. 18457994 10.1016/j.it.2008.03.002

[15] Chen R, Sigdel TK, Li L, Kambham N, Dudley JT, et al (2010) Differentially expressed RNA from public microarray data identifies serum protein biomarkers for cross-organ transplant rejection and other conditions. PLoS Comput Biol 6. 10.1371/journal.pcbi.1000940 PMC2944782 20885780

[16] Bromberg JS, Ikle D (2012) Is the time ripe for genomic diagnosis and prediction of rejection? Am J Transplant 12: 2573–2574. 23009138 10.1111/j.1600-6143.2012.04250.x PMC3967409

[17] Purvesh Khatri SR, Kimura N, De Vusser K, Morgan AA, Gong Y, et al (2013) A common rejection module (CRM) for acute rejection across multiple organs identifies novel therapeutics for organ transplantation. JEM 10.1084/jem.20122709 PMC3804941 24127489

[18] Billingham ME, Cary NR, Hammond ME, Kemnitz J, Marboe C, et al (1990) A working formulation for the standardization of nomenclature in the diagnosis of heart and lung rejection: Heart Rejection Study Group. The International Society for Heart Transplantation. The Journal of heart transplantation 9: 587–593. 2277293

[19] St Goar FG, Pinto FJ, Alderman EL, Valantine HA, Schroeder JS, et al (1992) Intracoronary ultrasound in cardiac transplant recipients. In vivo evidence of “angiographically silent” intimal thickening. Circulation 85: 979–987. 1537134 10.1161/01.cir.85.3.979

[20] Schmittgen TD, Livak KJ (2008) Analyzing real-time PCR data by the comparative C(T) method. Nat Protoc 3: 1101–1108. 18546601 10.1038/nprot.2008.73

[21] Sahai H, Khurshid A (1995) A note on confidence intervals for the hypergeometric parameter in analyzing biomedical data. Comput Biol Med 25: 35–38. 7600759 10.1016/0010-4825(95)98883-f

[22] Ferreira JA (2007) The Benjamini-Hochberg method in the case of discrete test statistics. Int J Biostat 3 Article 11. 10.2202/1557-4679.1065 22550651

[23] Shen-Orr SS, Tibshirani R, Khatri P, Bodian DL, Staedtler F, et al (2010) Cell type-specific gene expression differences in complex tissues. Nat Methods 7: 287–289. 20208531 10.1038/nmeth.1439 PMC3699332

[24] Alpert S, Lewis NP, Ross H, Fowler M, Valantine HA (1995) The relationship of granzyme A and perforin expression to cardiac allograft rejection and dysfunction. Transplantation 60: 1478–1485. 8545878 10.1097/00007890-199560120-00019

[25] Baan CC, van Emmerik NE, Balk AH, Quint WG, Mochtar B, et al (1994) Cytokine mRNA expression in endomyocardial biopsies during acute rejection from human heart transplants. Clin Exp Immunol 97: 293–298. 8050179 10.1111/j.1365-2249.1994.tb06083.x PMC1534709

[26] de Groot-Kruseman HA, Baan CC, Hagman EM, Mol WM, Niesters HG, et al (2002) Intragraft interleukin 2 mRNA expression during acute cellular rejection and left ventricular total wall thickness after heart transplantation. Heart 87: 363–367. 11907013 10.1136/heart.87.4.363 PMC1767064

[27] Shulzhenko N, Morgun A, Zheng XX, Diniz RV, Almeida DR, et al (2001) Intragraft activation of genes encoding cytotoxic T lymphocyte effector molecules precedes the histological evidence of rejection in human cardiac transplantation. Transplantation 72: 1705–1708. 11726838 10.1097/00007890-200111270-00025

[28] Shulzhenko N, Morgun A, Rampim GF, Franco M, Almeida DR, et al (2001) Monitoring of intragraft and peripheral blood TIRC7 expression as a diagnostic tool for acute cardiac rejection in humans. Hum Immunol 62: 342–347. 11295466 10.1016/s0198-8859(01)00211-7

[29] Shulzhenko N, Morgun A, Franco M, Souza MM, Almeida DR, et al (2001) Expression of CD40 ligand, interferon-gamma and Fas ligand genes in endomyocardial biopsies of human cardiac allografts: correlation with acute rejection. Braz J Med Biol Res 34: 779–784. 11378668 10.1590/s0100-879x2001000600013

[30] van Emmerik N, Baan C, Vaessen L, Jutte N, Quint W, et al (1994) Cytokine gene expression profiles in human endomyocardial biopsy (EMB) derived lymphocyte cultures and in EMB tissue. Transpl Int 7 Suppl 1: S623–626. 11271323 10.1111/j.1432-2277.1994.tb01458.x

[31] Kimball PM, Radovancevic B, Isom T, Spickard A, Frazier OH (1996) The paradox of cytokine monitoring-predictor of immunologic activity as well as immunologic silence following cardiac transplantation. Transplantation 61: 909–915. 8623159 10.1097/00007890-199603270-00012

[32] Lagoo AS, George JF, Naftel DC, Griffin AK, Kirklin JK, et al (1996) Semiquantitative measurement of cytokine messenger RNA in endomyocardium and peripheral blood mononuclear cells from human heart transplant recipients. J Heart Lung Transplant 15: 206–217. 8672525

[33] Morgun A, Shulzhenko N, Diniz RV, Almeida DR, Carvalho AC, et al (2001) Cytokine and TIRC7 mRNA expression during acute rejection in cardiac allograft recipients. Transplant Proc 33: 1610–1611. 11267440 10.1016/s0041-1345(00)02613-0

[34] Sarwal M, Chua MS, Kambham N, Hsieh SC, Satterwhite T, et al (2003) Molecular heterogeneity in acute renal allograft rejection identified by DNA microarray profiling. N Engl J Med 349: 125–138. 12853585 10.1056/NEJMoa035588

[35] Khatri P, Sarwal MM (2009) Using gene arrays in diagnosis of rejection. Curr Opin Organ Transplant 14: 34–39. 19337144 10.1097/MOT.0b013e32831e13d0

[36] Horwitz PA, Tsai EJ, Putt ME, Gilmore JM, Lepore JJ, et al (2004) Detection of cardiac allograft rejection and response to immunosuppressive therapy with peripheral blood gene expression. Circulation 110: 3815–3821. 15583081 10.1161/01.CIR.0000150539.72783.BF

[37] Pham MX, Teuteberg JJ, Kfoury AG, Starling RC, Deng MC, et al (2010) Gene-expression profiling for rejection surveillance after cardiac transplantation. N Engl J Med 362: 1890–1900. 20413602 10.1056/NEJMoa0912965

[38] Solez K, Axelsen RA, Benediktsson H, Burdick JF, Cohen AH, et al (1993) International standardization of criteria for the histologic diagnosis of renal allograft rejection: the Banff working classification of kidney transplant pathology. Kidney Int 44: 411–422. 8377384 10.1038/ki.1993.259

[39] Stewart S, Fishbein MC, Snell GI, Berry GJ, Boehler A, et al (2007) Revision of the 1996 working formulation for the standardization of nomenclature in the diagnosis of lung rejection. J Heart Lung Transplant 26: 1229–1242. 18096473 10.1016/j.healun.2007.10.017

[40] Wu T, Abu-Elmagd K, Bond G, Nalesnik MA, Randhawa P, et al (2003) A schema for histologic grading of small intestine allograft acute rejection. Transplantation 75: 1241–1248. 12717210 10.1097/01.TP.0000062840.49159.2F

[41] Delgado DH, Luu L, Edwards J, Cardella C, Rao V, et al (2002) Should moderate acute rejection of a cardiac transplant graft be treated? Clin Transplant 16: 217–221. 12010147 10.1034/j.1399-0012.2002.01132.x

[42] Fishbein MC, Bell G, Lones MA, Czer LS, Miller JM, et al (1994) Grade 2 cellular heart rejection: does it exist? J Heart Lung Transplant 13: 1051–1057. 7865512

[43] Nielsen H, Sorensen FB, Nielsen B, Bagger JP, Thayssen P, et al (1993) Reproducibility of the acute rejection diagnosis in human cardiac allografts. The Stanford Classification and the International Grading System. J Heart Lung Transplant 12: 239–243. 8476896

[44] Winters GL, McManus BM (1996) Consistencies and controversies in the application of the International Society for Heart and Lung Transplantation working formulation for heart transplant biopsy specimens. Rapamycin Cardiac Rejection Treatment Trial Pathologists. J Heart Lung Transplant 15: 728–735. 8820790

[45] Yeoh TK, Frist WH, Eastburn TE, Atkinson J (1992) Clinical significance of mild rejection of the cardiac allograft. Circulation 86: II267–271. 1424011

[46] Stewart S, Winters GL, Fishbein MC, Tazelaar HD, Kobashigawa J, et al (2005) Revision of the 1990 working formulation for the standardization of nomenclature in the diagnosis of heart rejection. J Heart Lung Transplant 24: 1710–1720. 16297770 10.1016/j.healun.2005.03.019

[47] Laguens RP, Meckert PM, Martino JS, Perrone S, Favaloro R (1996) Identification of programmed cell death (apoptosis) in situ by means of specific labeling of nuclear DNA fragments in heart biopsy samples during acute rejection episodes. J Heart Lung Transplant 15: 911–918. 8889987

[48] Bernstein D, Williams GE, Eisen H, Mital S, Wohlgemuth JG, et al (2007) Gene expression profiling distinguishes a molecular signature for grade 1B mild acute cellular rejection in cardiac allograft recipients. J Heart Lung Transplant 26: 1270–1280. 18096478 10.1016/j.healun.2007.09.017

[49] Holweg CT, Potena L, Luikart H, Yu T, Berry GJ, et al (2011) Identification and classification of acute cardiac rejection by intragraft transcriptional profiling. Circulation 123: 2236–2243. 21555702 10.1161/CIRCULATIONAHA.109.913921 PMC3115694

[50] Tusher VG, Tibshirani R, Chu G (2001) Significance analysis of microarrays applied to the ionizing radiation response. Proc Natl Acad Sci U S A 98: 5116–5121. 11309499 10.1073/pnas.091062498 PMC33173

[51] Russell ME, Fujita M, Masek MA, Rowan RA, Billingham ME (1993) Cardiac graft vascular disease. Nonselective involvement of large and small vessels. Transplantation 56: 1599–1601. 8279051

[52] Stoica SC, Cafferty F, Pauriah M, Taylor CJ, Sharples LD, et al (2006) The cumulative effect of acute rejection on development of cardiac allograft vasculopathy. J Heart Lung Transplant 25: 420–425. 16563972 10.1016/j.healun.2005.11.449

[53] Hornick P, Smith J, Pomerance A, Mitchell A, Banner N, et al (1997) Influence of acute rejection episodes, HLA matching, and donor/recipient phenotype on the development of ‘early’ transplant-associated coronary artery disease. Circulation 96: II-148–153. 9386090

[54] Yamani MH, Taylor DO, Rodriguez ER, Cook DJ, Zhou L, et al (2007) Transplant vasculopathy is associated with increased AlloMap gene expression score. J Heart Lung Transplant 26: 403–406. 17403484 10.1016/j.healun.2006.12.011

